# Trends and Innovations in Wearable Technology for Motor Rehabilitation, Prediction, and Monitoring: A Comprehensive Review

**DOI:** 10.3390/s24247973

**Published:** 2024-12-13

**Authors:** Pedro Lobo, Pedro Morais, Patrick Murray, João L. Vilaça

**Affiliations:** 1 2AI, School of Technology, IPCA, 4750-810 Barcelos, Portugal; pmorais@ipca.pt (P.M.); jvilaca@ipca.pt (J.L.V.); 2 LIFE Research Institute, TUS—Technological University of the Shannon, V94 EC5T Limerick, Ireland; patrick.murray@tus.ie; 3 LASI—Associate Laboratory of Intelligent Systems, 4800-058 Guimarães, Portugal

**Keywords:** wearable, continuous health, telemedicine, upper limb, physiotherapy, pos-strock, Parkinson’s

## Abstract

(1) Background: Continuous health promotion systems are increasingly important, enabling decentralized patient care, providing comfort, and reducing congestion in healthcare facilities. These systems allow for treatment beyond clinical settings and support preventive monitoring. Wearable systems have become essential tools for health monitoring, but they focus mainly on physiological data, overlooking motor data evaluation. The World Health Organization reports that 1.71 billion people globally suffer from musculoskeletal conditions, marked by pain and limited mobility. (2) Methods: To gain a deeper understanding of wearables for the motor rehabilitation, monitoring, and prediction of the progression and/or degradation of symptoms directly associated with upper-limb pathologies, this study was conducted. Thus, all articles indexed in the Web of Science database containing the terms “wearable”, “upper limb”, and (“rehabilitation” or “monitor” or “predict”) between 2019 and 2023 were flagged for analysis. (3) Results: Out of 391 papers identified, 148 were included and analyzed, exploring pathologies, technologies, and their interrelationships. Technologies were categorized by typology and primary purpose. (4) Conclusions: The study identified essential sensory units and actuators in wearable systems for upper-limb physiotherapy and analyzed them based on treatment methods and targeted pathologies.

## 1. Introduction

The theme of telemedicine through the adoption of wearables has seen a significant increase in recent years. Through a quick search on scientific research indexing platforms such as Web of Science using the keywords ‘telemedicine’ and ‘wearable’, we have observed a steady growth in published papers from 2010 to 2023.

While there was already a gradual increase in publications on this topic till 2019, largely due to the increasing adoption of smartwatches and smart bands, from 2020 onward, we can observe an exponential surge in publications due to the COVID-19 pandemic. This surge is primarily attributed to the issues associated with the pandemic, such as the need for social distancing, the isolation of high-risk patients, and the challenges in delivering healthcare during periods of high demand, driven by centralized facilities and limitations in both human and non-human resources. This observation is also substantiated in some of the most recent literature review articles [1,2,3].

While it has been a sporadic issue in some countries that has gradually subsided over time, we currently live in a century where one of the main challenges is aging and has been a cause for concern in many studies. Globally, in 2019, Europe and North America had the highest percentage of aged population, with 18 percent of the population being 65 years or older. The trend of an increasing aging population, in particular in Europe, is expected to continue. The number of individuals aged 80 years or older is growing at an even faster rate. Specifically, in 1990, there were 54 million people aged 80 years or older globally, and by 2019, this number had nearly tripled to 143 million [4].

As widely known, aging is directly linked to the deterioration of individuals’ physical and cognitive functioning and their propensity to rely on conventional healthcare methods [5,6]. With this issue in mind, it is imperative to take action and allocate more efforts to the field of Remote Healthcare in order to avoid overwhelming traditional healthcare systems as we currently know them. By further implementing Remote Healthcare, we can not only provide closer and personalized monitoring of individuals but also use it continuously throughout people’s lives as an excellent tool for the prevention and early detection of health problems.

In recent years, there has been a noticeable absence of scientific publications of literature reviews in the field of the motor rehabilitation, prediction, and monitoring of pathologies directly associated with motor limitations in the upper limbs. While there have been comprehensive reviews of wearables used in high-performance sports for monitoring physiological developments and injury prediction, showcasing the potential of technology, this attention has not been adequately directed towards physiotherapeutic applications [7]. The existing literature covers various areas, such as wearable-based sleep monitoring, cardiac monitoring [8,9,10,11,12], materials with sensory capabilities [13,14,15,16], wearable communication [17,18,19,20], and physical activity [21,22,23].

However, the exploration of tracking human movement remains limited. For instance, Lou et al. [24], focused on flexible sensors, provides only a small section of solutions capable of monitoring the motor activity of the upper limbs. Bortolani et al. [25] conducted an extensive literature review aiming to identify all Test of Motor Function (TOM) assessments used for evaluating motor function in patients with neuromuscular diseases. The study focused on a descriptive summary of the technological aspects employed in these assessments and assessing the available evidence regarding psychometric properties. The analysis included only 100 studies due to restrictive filtering criteria, which required the inclusion of patients with clearly identified neuromuscular diseases.

According to recent data, despite the fact that approximately 25% of commercialized wearables and 30% of research-targeted wearables are related to physical activity [26], no literature reviews specifically addressing this category of devices have been identified in the past four years. The last comprehensive review addressing this situation dates to 2019 with the work of Elvira et al. [27]. Hence, there is a pressing need to develop a literature review on the utilization of wearables in the rehabilitation, monitoring, and prediction of the motor progression and/or degradation of the upper limb.

The proposed study aims to examine wearable systems for the purpose of motor rehabilitation, monitoring, and prediction. During our review, we identified two primary categories of works, fundamental technologies and associated technologies. Fundamental technologies were further divided into three interconnected subgroups, accessories, exosuits, and exoskeletons, and one independent group, cameras. We also identified a small percentage of studies that did not fit into these two categories, which were grouped under a separate “others” section. Our focus will be on accessories, as they represent the most prominent continuous health promotion systems today. Cameras, despite being less represented, were included due to their relevance in telemonitoring.

The structure of the paper is outlined as follows. The abbreviations presented throughout the paper are introduced in Section 2. Section 3 introduces the methodology employed for the systematic review. In Section 4, wearable systems are categorized and described. The discussion of the key findings is presented in Section 5. Lastly, Section 6 provides the main conclusions and future perspectives.

## 2. Acronyms

In this work, we utilize different acronyms and truncations for specialized terms and ideas broadly recognized within the pertinent areas. To encourage perusing and reference all through the article, we display below a list of all the acronyms utilized, at the side their definitions (see Table 1). The purpose of this list is to help the reader in understanding the substance more clearly, without overstating the total terms within the content. We energize perusers to allude to this segment at whatever point fundamental amid their perusing.

## 3. Methods

### 3.1. Search Strategy

In this section, we outline the approach employed in conducting the current systematic review, detailing the search strategy and criteria applied to include the chosen papers.

Was conducted a computer-assisted search on the Web of Science database using the search terms: “wearable” and “upper-limb” and (“rehabilitation” or “monitor” or “predict”). This search encompassed the timeframe from January 2019 to December 2023. All titles and corresponding abstracts identified through the search terms were reviewed, and articles meeting the selection criteria underwent a thorough detailed reading and examination.

### 3.2. Selection Criteria

From the published articles obtained through the aforementioned search strategy, a set of criteria was defined to identify the relevant works for this review analysis. First, review articles were automatically excluded.

Among the remaining works, all papers featuring devices aimed solely at lower-limb health, often associated with gait, were removed due to being out of scope. Documents categorized as “Abstracts only” and those working with amputee prostheses were also not considered. Some studies testing the reliability of devices for acquiring physiological data specifically in the upper limb, but with no impact on mobility quality, were also excluded. Devices designed for industrial purposes and works that promoted physical activity or performance without a physiotherapeutic purpose were also removed. Studies focusing on the sensory aspect, particularly the material aspect as well as musical cueing, and any research addressing pathologies unrelated to the degradation of upper-limb mobility, were not included in the analysis. Ultimately, all studies that encompass technologies of the exoskeletons and exosuits typology were not considered.

### 3.3. Search Results

**Figure 1:**
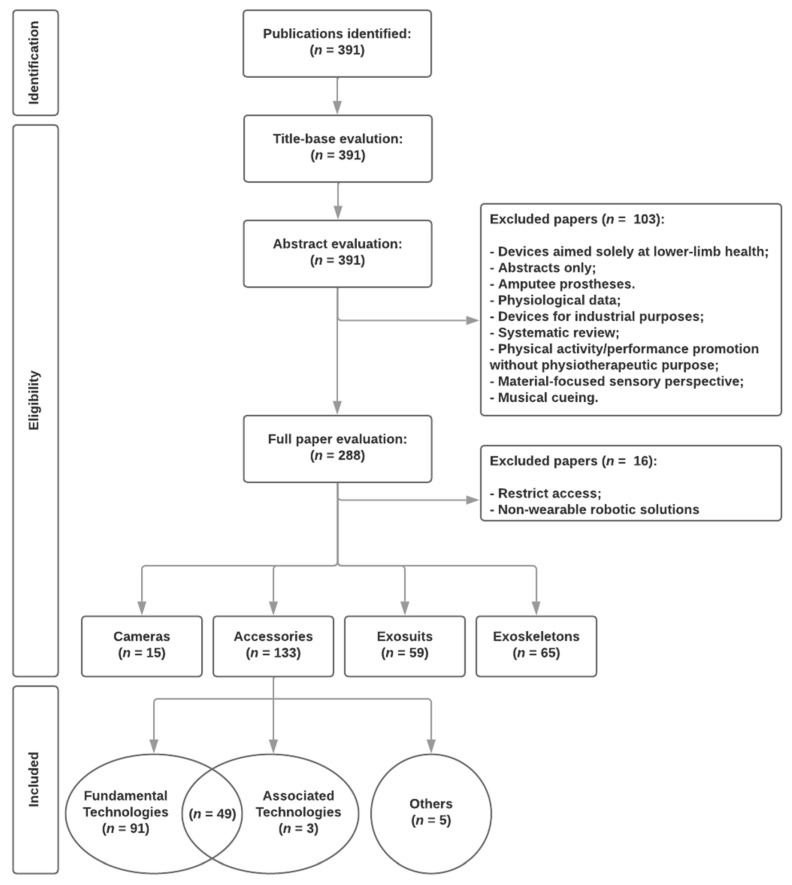
The data flow of the systematic review.

The search in the databases, using the strategy previously presented, resulted in 391 publications. Upon abstract evaluation, 103 papers were further rejected for not meeting the specified selection criteria. These papers did not directly or indirectly address the use of wearable devices in a physiotherapeutic context, focusing on monitoring and predicting the evolution and/or degradation of upper-limb mobility. Following the exclusion of works with restricted access, non-wearable robotic solutions, exosuits, and exoskeletons, 148 papers underwent full-text analysis, forming the basis for exploration in this review. The included works were organized into 3 conceptual groups: fundamental technologies, associated technologies, and others. The systematic review’s data flow is illustrated in Figure 1.

## 4. Results

The utilization of wearables in monitoring and rehabilitating the upper limb has emerged as an innovative approach for various health conditions. Throughout the analysis of the included studies, various health conditions were investigated. However, one condition is prevalent—post-stroke rehabilitation, which was mentioned in approximately 43% of the studies. In second place, Parkinson’s disease (PD) emerges with 7% of mentions.

Numerous investigations focused on overall upper-limb rehabilitation, while others explored a range of conditions, such as spinal cord injuries (SCI), arthritis, dyspraxia, ataxia, neonatal brachial plexus palsy, sclerosis, spasticity, cerebral palsy (CP), dyskinesia, and various joint injuries and post-operative progressions in upper-limb motor function. The interrelated character of various health issues, especially among post-stroke individuals, was considered.

### 4.1. Overview

**Figure 2:**
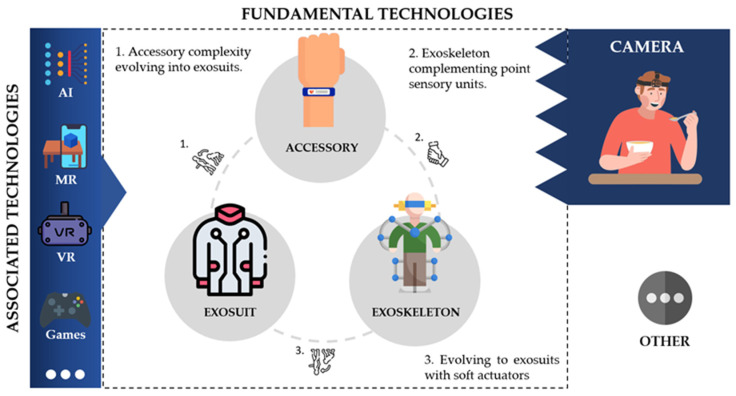
The figure overview of the technological groups identified throughout our review as well as the relationship between them.

In addressing the challenges outlined in the aforementioned analyses, several technologies were studied and developed in these works. The included articles were divided into two main categories: fundamental technologies and associated technologies. Additionally, a small percentage of works that did not fit into these categories were grouped under “others” (see Figure 2). We defined fundamental technologies as devices equipped with sensor and/or actuating units selectively applied on the patient, such as wristbands, rings, skin-adherent sensors, among others. Within this category, they can also be viewed as active or passive: passive when they are sensory devices and active when they include actuators to apply stimuli to individuals. In the case of cameras, they do not relate significantly to the technological groups presented thus far, playing a predominant role in analyzing upper-limb object manipulation by patients and providing information to healthcare professionals. Given the significance of these types of devices and the wide range of solutions identified in the literature, they will be explored in more detail in Section 4.2.

In the case of camera work, the described works mostly served as a monitoring tool and to support healthcare professionals in diagnosing the functioning of the upper limb. Many of the works used cameras to acquire egocentric vision or first-person vision, which were attached to patients. In the field of egocentric videos, Zariffa et al. has contributed with 10 works focused on the concept of egocentric cameras [28,29,30,31,32,33,34,35,36,37]. The underlying concept involves the continuous monitoring of patients’ hand usage and their interaction with everyday objects (see a conceptual scheme in Figure 2). Throughout the various works presented, many employed deep learning (DL) and machine learning (ML) methods to achieve this monitoring, utilizing object detectors [29,30,31,32,33,34,35,36] (such as Yolo) and OpenPose [32], for example. This approach enables the provision of feedback to healthcare professionals about patient behavior, facilitating clinical decision-making. Although some are tailored to different pathologies and contexts, they share a similar principle. Moreover, Battraw et al. [38] presented a similar concept of utilizing an egocentric camera, with a particular focus on pediatric cases, to identify grasp configurations in children.

Outside the concept of egocentric vision, Ham et al. [39] developed a mixed reality (MR) board as an upper-extremity training tool for stroke patients, sensing hand movements using a deep camera and tangible objects for upper-extremity rehabilitation within an MR environment. In Ozgur et al. [40]’s work, the authors proposed a technological solution to detect/correct compensatory movements during physiotherapy that can reduce its effectiveness. For that, a Tangible Robot-Assisted system was proposed. First, a vision system was developed to detect the presence of compensatory movements in this treatment. After confirming that patients indeed developed compensatory movements during treatment, the authors proposed incorporating an IMU into the Tangible Robot to achieve the real-time detection of these movements.

**Figure 3:**
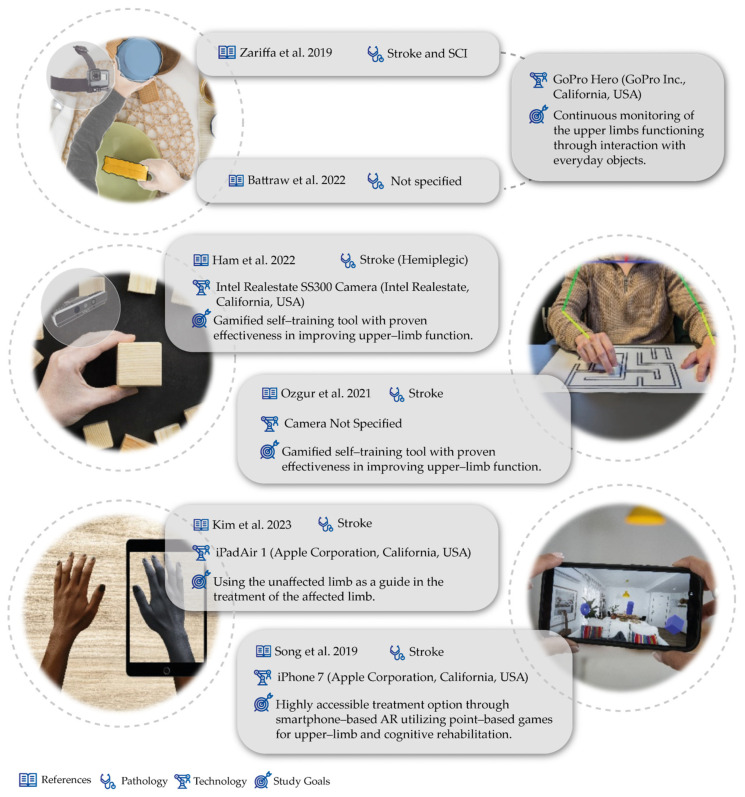
Study characteristics for camera studies [28,29,30,31,32,33,34,35,36,37,38,39,40,41,42].

Moreover, using cameras, Kim et al. [41] presented a work about video augmented mirror therapy. This work involved capturing images of each participant’s unaffected upper extremity and then left–right reversing them using the Video Rotate and Flip application (Wander Bit LLC) to create images of the participant’s affected upper extremity during reach-to-grasp treatments. The results showed improvements in the motor control of reach-to-grasp kinematics and upper-extremity function compared to traditional mirror therapy and conventional rehabilitation. A different perspective was presented by Song et al. [42], which developed a work focused on the use of augmented reality (AR) through the functionality of a smartphone camera. Specifically, they created an AR game involving placing virtual objects in the patient’s surrounding environment, where the patient can interact with them by moving the smartphone with the affected limbs toward the virtual object (see Figure 3).

**Figure 4:**
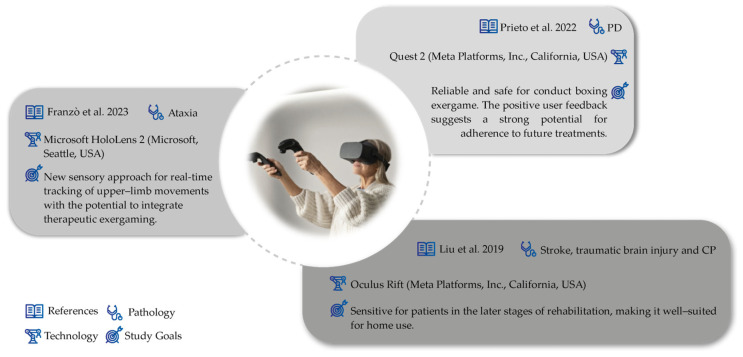
Study characteristics for complementary technologies studies [43,44,45].

Regarding associated technologies, we consider them to be an extension of fundamental technologies. Although certain associated technologies may independently hold relevance for physiotherapeutic purposes, our review focuses primarily on studies that leverage the potential of fundamental technologies through their integration with associated technologies. In these cases, fundamental technologies are viewed largely as tools that enhance the capabilities of associated technologies. Within this category, we find virtual reality (VR), AR, and MR often associated with gamifying physiotherapeutic processes, in which wearables are frequently used as game controllers. Additionally, we frequently identify the implementation of artificial intelligence (AI) methods in data processing to monitor and predict patients’ motor evolution. Within this concept, there are some studies that explore these ideas in relation to wearables. In the concept of VR, we have observed initial usability studies of the Oculus Quest 2 and games for physiotherapy in patients with Parkinson’s [43]. Additionally, effective applications in treatment through tracking upper-limb extremities during exercises in a game format were also described [44]. Furthermore, a work focused on the symbiosis between VR and a non-wearable haptic feedback system (Omega.7, Force Dimension) was also presented. This work consisted of a maze-like game where the user used a haptic feedback device to guide the ball out of it, involving virtual guiding tasks with haptic feedback to assess wrist motor functions, including basic motor flexibility, motion stability, and the range of active motion (see Figure 4) [45].

**Figure 5:**
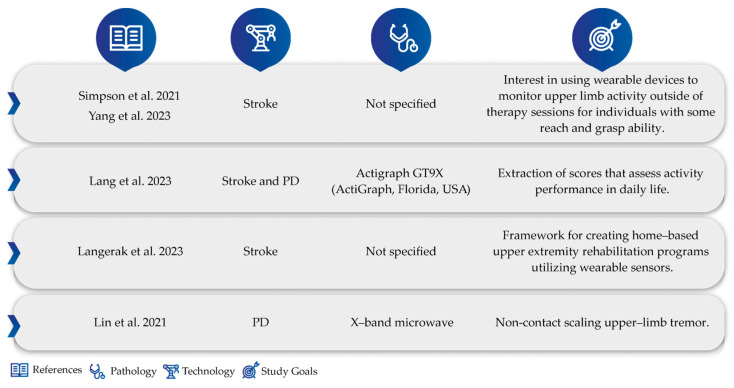
Study characteristics for other studies [46,47,48,49,50].

Finally, in the “other” group, we encounter studies that do not focus on any specific technology but are nonetheless relevant to the wearable theme in terms of motor rehabilitation, monitoring, and prediction. These studies focus on wearable design preferences, feedback from patients/physicians regarding implementation, and the identification of physiotherapy-relevant exercises, among others, such as Simpson et al.‘s work [46]. This team conducted a qualitative study carried out by healthcare professionals on the potential of wearable devices for capturing upper-limb activity post-stroke. The conclusion was unanimous, showing interest in using wearable devices to capture upper-limb activity outside of therapy sessions for individuals with some reach and grasp ability. Also, from a qualitative perspective, both by healthcare professionals and individuals’ post-stroke, researchers also conducted studies to determine essential considerations for designing and developing an interactive wearable system for upper-extremity rehabilitation [47]. On the other hand, in Lang et al. [48]’s work, the direct effects of physiotherapy on patients’ daily lives were studied. Through the longitudinal monitoring of capacity and performance via wearable sensor measurements of use in ratio/steps/day, it was found that the capacity acquired by patients with clinic treatments did not directly translate into better performance in daily life. It was observed that performance does not improve proportionally to capacity. Furthermore, looking at the potential of wearable motion sensors, Langerak et al.‘s study [49] raised the types of exercises to be performed at home, subdivided into functional requirements, required exercises, and exercise measures. The requirements are prioritized as must-haves, should-haves, and could-haves. They can be used to develop home-based UE rehabilitation interventions based on wearable motion sensors. Still in the “other” group, we must mention Lin et al.‘s work [50]. This study started from the premise that non-contact measurement devices can also digitize handwritten patterns as well as wearables commonly associated with direct contact tremor measurements. In their work, they presented a non-contact measurement with an array X-band microwave (10 GHz) Doppler-based linear quantizer designed to continuously measure upper-limb movements for tremor class scaling. They extracted the physical changes in the oscillation frequencies, amplitudes, and directions of tremor signals for scaling upper-limb tremor (ULT) levels. In experiments involving 10 subjects, the proposed non-contact bioradar sensor could quantify asymmetrical and irregular oscillations (see Figure 5).

### 4.2. Accessories

Numerous devices have been covered in this review, ranging from various sensor units to actuators, presenting a diverse array of applications. In exploring sensor units, a wide range of typologies have been investigated, including inertial measurement units (IMUs), electromyography (EMG), force myography (FMG), mechanomyography (MMG), barometric pressure sensors, A-mode ultra sound sensors, force-sensitive resistive (FSR) sensors, bend and pressure sensors, proximity-based sensors, electroencephalographic (EEG) and electrooculographic (EoG) sensors, and physiological data sensors, such as heart rate variability (HRV) and skin conductance level (SCL) sensors. Additionally, sensors like LED/phototransistors, strain sensors, electrocardiography (ECG), galvanic skin responses (GSR), skin temperature (SKT) sensors, and radio frequency identification (RFID) are explored. Notably, IMUs are prominently featured in approximately 66% of wearable-related studies, followed by EMG at around 25%. While other sensors receive fewer mentions, their significance is not diminished, and quantifying their presence proves less relevant in comparison.

Regarding actuators, only two types emerged: vibration or electrical stimulation. Out of the 17 studies addressing accessory-type wearables, only 5 utilize electrical stimulation as an active mechanism.

#### 4.2.1. IMUs

IMUs emerge as the most widely used sensor, highlighting their versatility for multiple purposes. This sensor was used in a high number of different applications, largely associated with users’ reach capabilities. As will be presented further, the potentialities of these technologies are vast, with numerous works exploring applications in real-world problems. Additionally, studies have emerged addressing specific technical issues, such as the requirements of devices incorporating these technologies for certain pathologies or scenarios as well as the establishment of physiotherapy. Brown et al. [51] conducted a study about how a wristband should be made to encourage movement of the affected upper limb in unilateral cerebral palsy. Turk et al. [52] developed a comprehensive collection of upper-limb tasks tailored for the M-MARK wearable system aimed at offering real-time feedback during daily tasks for individuals recovering from strokes. Through consultations with rehabilitation experts and interviews with stroke survivors, data were gathered to inform the selection process. Subsequently, employing a categorization matrix, they methodically pinpointed 11 training tasks considered appropriate for evaluation with the M-MARK system.

In line with Lang et al. [48], addressing the issue of the lack of direct impact of clinic-based physiotherapy on specific daily movements, David et al.‘s work [53] highlighted this same target in their work. In this work, the issue of the lack of direct impact of clinic-based physiotherapy on specific daily movements, such as opening a bag, was highlighted. They proposed a framework based on wearable IMUs to characterize daily movements and adjust physiotherapy accordingly. In particular, this study is an example of home-based physiotherapy by post-stroke patients [54].

There was still a study that proposed non-IMU sensors but with similar objectives. Zhang et al. [55] proposed a wristband with an airflow sensor to track arm movements by varying the airflow, aiming to quantitatively evaluate energy expenditure during post-stroke rehabilitation.

Within IMU sensory typology, it was possible to categorize the works into 6 conceptual groups: (i) monitor, diagnostic, and motor dysfunction assessment; (ii) comparison with other methods; (iii) machine learning; (iv) gamified rehabilitation; and (v) post-operative rehabilitation.

i. Monitor, Diagnostic, and Motor Dysfunction Assessment:

IMUs were used to assess asymmetry and activity differences between affected and non-dominant hands in post-stroke patients with right hemiparesis [56]. In Beani et al.’s study [57], using the ActiGraph GT3X+ (ActiGraph, Pensacola, FL, USA), asymmetry in the use of the two upper limbs in children with unilateral cerebral palsy was measured.

Inspired by the same concept, Hughes et al. used a low-cost single IMU sensor-based wearable system (outREACH) to determine upper-limb impairment [58]. In this study, participants performed an object manipulation task with the affected and unaffected limb, and the sensor was sensitive to differences in performance-based upper-limb impairment.

**Figure 6:**
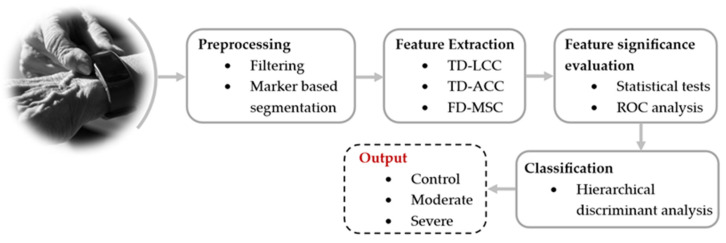
Identifying hemiparesis using wrist-worn accelerometry, as presented in the work by S. Datta et al. [59].

In post-stroke patients, Datta et al. demonstrated that wrist devices also proved effective in analyzing upper-limb movements and identifying hemiparesis in acute stroke patients. Moreover, these wrist devices distinguished between controls and moderate-to-severe hemiparesis [59] (see Figure 6). In another of his works, Datta et al. [60] demonstrated the feasibility of identifying the severity of hemiparesis in acute stroke cases using IMUs based on a bivariate Poincaré plot during bilateral hand activity. Additionally, it was demonstrated that four classes of hemiparesis severity in acute stroke can be identified from short-length wearable accelerometry using only one sensor worn on each wrist [61,62].

An assessment of upper-limb functionality involves the use of wearable wrist sensors, such as accelerometers, to track and measure upper-limb gross movement (GM) in children diagnosed with CP [63]. This technology also served to assign a gross movement score to quantify arm use in patients with hemiparesis [64].

A comparative study of the number of reach and grasp in post-stroke patients and healthy individuals was conducted, using a wristband (TENZR). By detecting movements of the upper limbs (IMU) combined with volumetric changes in the fingers and wrist (Force Myography—FMG), it was possible to count the repetitions of the reach and grasp activity [65]. Also, to consistently capture functional reach-to-grasp repetitions, Simpson et al. [66] combined IMU, FMG, and a proximity sensor for mild-to-moderate upper-limb stroke impairment. Schwarz et al. [67] analyzed reach-to-grasp tasks in chronic stroke patients using wearable sensors to examine trunk compensation and joint movement correlations with clinical impairment.

From the perspective of monitoring physiotherapeutic activities, Palani et al. [68] developed a portable and economical platform that integrates IMU and vision data to accurately estimate joint angles in real time during rehabilitation tasks. Data fusion is enhanced by a Kalman filter, providing efficiency and accuracy according to the authors. Although for a different purpose, Humadi et al. [69] applied inertial measurement units for joint angle measurement in the field during manual handling tasks for ergonomic risk assessment. Still in the field of joint angle measurements, Rahman et al. [70] implemented a Madgwick filter-based joint angle measurement algorithm to build a wireless wearable sensor system for simplified joint angle measurements. Rajkumar et al. [71] developed a sensor network to calculate joint angles of the shoulder and elbow using 5 IMUs.

**Figure 7:**
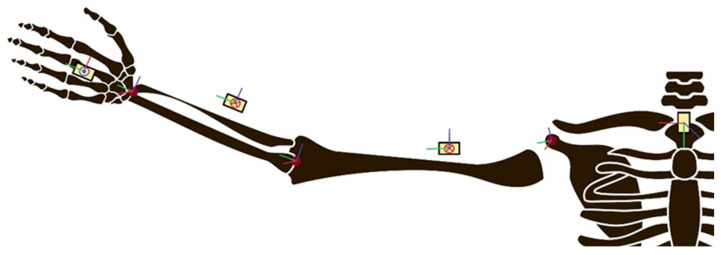
An illustration of the sensor network used for the reconstruction of upper-limb joints from the work of Meng et al. and the anatomical model of the entire upper limb with the definition of joint axes.

Using IMUs, an upper-limb range of motion (ROM) assessment was also conducted through a two-layer model, enabling the simultaneous estimation of joint angles and positions. This model addresses precision challenges by implementing a dynamic sensor-to-segment calibration method [72] (see Figure 7). Concerning ROM, Toh et al. [73] also conducted a study on the applicability of wristbands in arm ROM measurements, but in his case, they integrated them with an ecosystem with a telerehabilitation app, allowing for interactive therapy.

There were also studies that described simple algorithm approaches for the categorization of 14 Manual Activities of Daily Living (ADLs). Using gyroscope data from six IMUs located on the thumb, index finger, and wrist of both hands, it was possible to classify manual ADLs into five categories [74]. Sebastjan Šlajpah et al. [75], with a wearable sensory system combining IMU and EMG sensors, monitored upper-limb movements during ADLs through time-based and path-based segmentation for trajectory and muscle activity analysis, being able to distinguish between affected and unaffected limbs.

Franchi de Cavalieri et al. [76] explored the potential of wearable sensors to predict clinical assessment scores of infants’ motor activity through data acquired by accelerometers placed on infants’ wrists and trunk during playtime. They exploited the method of functional data analysis to implement new models combining quantitative data and clinical scales. With more specificity, Gatword et al. [77], utilizing the same technology, leveraged it for the quantification of the duration and magnitude of patient-initiated arm movements outside the clinical setting in cases of neonatal brachial plexus palsy.

Furthermore, metrics were developed to assess upper-limb compensation in tetraplegic SCI patients using wearable sensors [78]. These metric-graded redefined assessments of strength, sensibility, and prehension can be applied in clinical intervention studies to examine the presence of upper-limb compensation. In the realm of wearable technology development, Gao et al. [79] focused on the development of a new armband geared towards data transmissibility, gateway nodes, and wireless communication networks, mainly due to concerns regarding mobile medicine. Despite the potential of this technology, the human acceptance must be evaluated, whether by patients or healthcare professionals. Addressing this concern, Jung et al. [80] realized further studies. Accelerometers were utilized on the fingers as a wearable for testing. According to the questionnaires, acceptance was observed among post-stroke individuals and occupational therapists (OTs). The OTs could easily customize treatments based on the sensory data. Formstone et al. [81] combined inertial measurement and mechanomyography (MMG) in a system to quantify hand and wrist motor function.

For the diagnosis and monitoring of ataxia progression, Tran et al. [82] conducted a design, implementation, and feasibility study of a new multimodal system using Microsoft Kinect (Microsoft, Seattle, USA) and wearable sensors for the assessment of ballistic tracking in individuals. Concerning PD, numerous studies have been developed to address this concern. Yousef et al. [83] implemented accelerometer-based wearable device technology in monitoring upper-limb tremor detection in essential tremor patients. During the study, patterns regarding the amplitude and frequency of voluntary and involuntary vibrations were observed. Furthermore, efforts were made to not only monitor PD progression but also define the onset of manifestation on other works [84].

The reliability of wearable technologies for physiotherapeutic purposes, monitoring, and predicting the motor evolution of individuals was further validated by Lang et al. [85]. They followed 67 participants after their first stroke, measuring upper-limb performance with wearable sensors. The obtained results, based on inertial data, showed rapid initial improvement, stabilizing within 3–6 weeks post-stroke, suggesting early adaptation to daily activities before functional capacity stabilization. Von Gunten et al. [86], in their study, evaluated the feasibility of Action Observation Training (AOT) combined with sensor-based measurements in infants at high risk of Unilateral Spastic CP. Sensor data correlated significantly with hand function, suggesting wearable sensors’ potential to monitor upper-limb function during AOT for infants at high risk of Unilateral CP. Finally, Vanmechelen et al. [87] concluded that it is possible to detect pathological movements in individuals with Dyskinetic CP using a network of inertial sensors. In this study, jerk and acceleration/angular velocity was found to be significantly higher in the group with Dyskinetic CP (see Table 2).

ii. Comparison with Other Methods:

Some studies compared IMU technologies against conventional methods. Henschke et al. [89] compared a portable inertial sensor system with an optical motion analysis (Mocap) to measure shoulder kinematic parameters. The authors found that the overall agreement with the gold standard was low. Chan et al. [90] conducted a comparative study between an IMU and conventional clinical methods (optical motion capture) in measuring ROM, indicating acceptable accuracy for the IMU compared to the conventional method.

Tran et al. [91] redesigned an IMU sensor system known as the T’ena sensor and evaluated its ability to measure movement kinematics accurately during common post-stroke motor tasks against a gold-standard motion capture system. High agreement and correlation values were observed between both systems. Later, Hughes et al. [92], based on the same platform, validated the wearable for assessing movement quality and efficiency.

This technology was also tested against Microsoft Kinect for motion tracking, demonstrating similar performance in human motion tracking [93,94] (see Table 3).

iii. Machine Learning:

Oubre et al. [95] used wrist-worn IMU to objectively assess ataxia severity and differentiate between ataxia, Parkinson’s, and controls with high accuracy. In this specific case, data processing was performed using ML techniques. Additionally, Oubre et al. developed two more works. In the first [96], they estimated upper-limb impairment in stroke survivors using data from two wearable inertial sensors processed by an unsupervised clustering algorithm and a supervised regression model, which were used to estimate Fugl–Meyer Assessment (FMA) scores. In the second [97], stroke impairment severity from the performance of ADLs was studied using only the data obtained from a single wrist-worn inertial sensor.

Regarding PD, Mirelman et al. [98] evaluated the best inertial data (IMUs) to diagnose PD and determine the stage of the disease (upper vs lower limb) also through machine learning methods (the upper limb was better). We also see the complementarity of IMUs with ML in PD through quantifying stages of the pathology [99] or as a diagnostic aid [100]. In the study [99], three unsupervised learning algorithms were compared: k-means, self-organizing maps, and hierarchical clustering. Self-organizing maps achieved the best results. In the study [100], a genetically optimized random forest classifier was used for three different upper-limb movements, obtaining the highest accuracy in the alternating hand movement task. In addition to diagnosis, there was also an attempt to differentiate Parkinson’s patients from essential tremor patients using support vector machine classifiers [101].

Although not directly related to any motor pathology, Wiebe H.K. de Vries et al. [102] focused on load applied on the shoulders, which can serve to predict future problems. This work involved the classification (through the identification of the type of activity performed) of the load exerted on the shoulders associated with wheelchair use through machine learning methods. Four EMGs and five IMUs (three in person + two in wheelchairs) were used. It was concluded that the use of EMGs was unnecessary and, regarding IMUs, accuracy increased as the number of IMUs used in the classification increased.

Similarly, upper-limb pain does not directly reflect motor dysfunction; however, it can be an indicator. In this segment, there was an innovative study in the application of ML models to predict pain based on IMUs, in which the results showed that characteristics related to the smoothness of measurements presented a stronger correlation with pain [103].

As previously mentioned, devices classified as accessories complement exoskeletons often associated with motion intention prediction. Although IMUs are not the most frequent sensory units for this purpose, studies have been developed on the application of neural networks for joint prediction based on IMU sensors [104,105]. In the case of Little et al. [106], IMU data were crossed with EMG and stretch sensors to be equally processed through machine learning techniques for detecting elbow movement intention. In this study, it was concluded that decision-making with only IMU data was faster but less accurate. In a similar sensory combination to [106], Yang et al. [107], through a multimodal sensing system, incorporating IMUs, EMG, and MMG sensors, captured kinematic and physiological signals during reaching and placing tasks by five subjects. These data served as input for traditional regression models and deep learning models for training and testing. As a result, it was concluded that IMU sensors alone, with the proposed model, are sufficient for motion intention detection.

In the field of monitoring physiotherapeutic evolution over time, Panwar et al. [108] reformulated a convolutional neural network (CNN) to classify three movements (extension, flexion, and rotation) using data from a wristband.

The combination of these two (IMU and ML) was also used to estimate clinical scores, more specifically the Movement Disorder Society-Sponsored Revision of the Unified Parkinson’s Disease Rating Scale, Part III. Adans Dester et al. [109] demonstrated that wearable sensor data can accurately estimate clinical scores used to assess motor impairments and upper-limb movement quality. The Upper-limb FMA scale was employed to assess motor impairment severity, while the Functional Ability Scale (FAS) was used to evaluate movement quality.

Regarding telerehabilitation, a system capable of identifying and logging the variety and frequency of rehabilitation exercises performed by the individual was created [110]. The system utilizes a smartwatch and a smartphone application integrated with a machine learning algorithm. Additionally, the effectiveness of this home-based rehabilitation system was assessed through a prospective comparative study involving chronic stroke survivors.

Through the processing of acceleration data provided by double wristbands through a k-mean cluster, Barth et al. [111] discovered that the best characterization of movement performance should be categorized into five distinct levels illustrating the performance score.

Liu et al. [112] showed that accelerometer recordings obtained from the proposed body-networked sensor system composed of a finger-worn and a wrist-worn sensor can be used to estimate the amount of hand use during ADLs. On the other hand, Subash et al. [113], with the aim of measuring not only hand use but rather the entire upper limb, conducted a comparative study of different measures for assessing upper-limb use using wrist-worn inertial sensors, categorizing them into threshold activity counting, gross movement score, and machine learning. The research found that machine learning, specifically the intra-subject random forest model, performed best in detecting upper-limb use. Among traditional methods, a hybrid approach combining activity counting and gross movement score showed promise.

Ernesto et al. [114] also studied the use of machine learning algorithms to estimate clinical scores according to the FAS, reflecting upper-limb movement quality based on wearable sensor data. A random forest-based algorithm demonstrated a high correlation (R^2^ = 0.91) with clinicians’ assessments, offering potential for precise rehabilitation interventions in individuals with upper-limb motor impairments.

**Figure 8:**
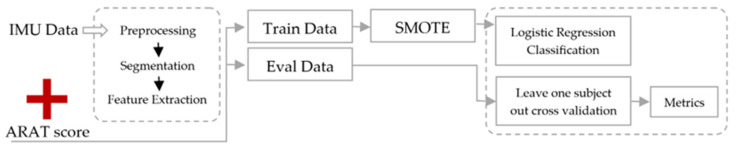
A flowchart of the methodology for estimating task-specific ARAT scores using inertial sensors mounted on the wrist. Synthetic minority over-sampling technique (SMOTE).

ML was also used to estimate task scores from the Action Research Arm Test (ARAT, conceptual blocks in Figure 8), one of the most widely used clinical tests of upper-limb motor functioning [115]. The data processed by the algorithm were from two wrist-worn inertial sensors, and an accuracy of 80% was achieved.

ML not only adds value to wearable devices with inertial units but also reinforced the benefits of their clinical applicability and complementarity with clinical data to predict and monitor the recovery process and assess the responsiveness to treatment on an individual basis. Lee et al. [116] concluded in their study that a clinical algorithm had a correlation of 0.79 with rehabilitation outcomes but failed to model variability in individual response. In contrast, the sensor algorithm had a correlation of 0.91 and modeled individual responses more accurately. By combining clinical and sensor data, a correlation of 0.94 was achieved.

**Figure 9:**
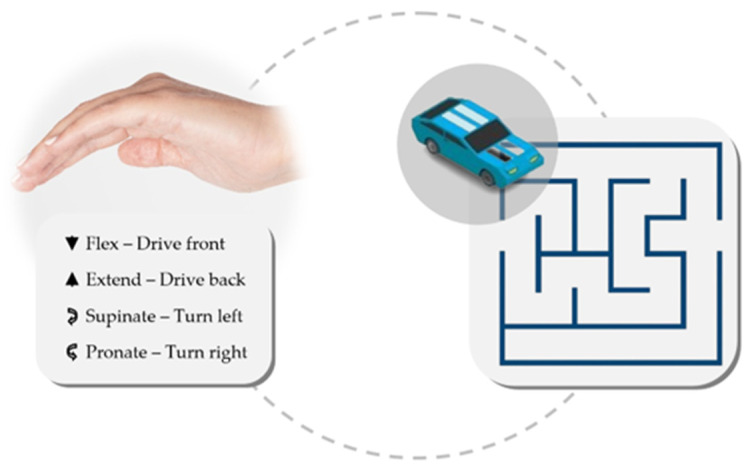
A wristband functions as a joystick for controlling a remote-controlled car within a maze; wrist flexion-extension controls the car’s forward and backward movement, while pronation–supination enables on-the-spot turning.

Within ML, comparative studies of the accuracy of multiple classifiers in a motion recognition method [117] and for the motion recognition of upper-limb exercises and improvement of recognition performance [118] were also conducted, always using data from IMUs. Regarding motion recognition, the optimal size of the time window for classifying real-time motions was also investigated [119]. This was achieved by utilizing CNN-based human activity recognition (HAR) with inertial data collected from a smartwatch (see Table 4).

iv. Gamified Rehabilitation:

The application of an accessory such as a gamepad for physiotherapeutic purposes was explored Segal et al. [120]. In this work, they employed IMU placed on the patient’s hand, aiming to control a cart within a maze based on the Yaw, Pitch, and Roll (YPR) coordinates. This approach aimed to enhance rehabilitation adherence by quantifying performance through game scores (see Figure 9). From a proprioceptive rehabilitation perspective, Lapresa et al. [121] explored a system employing knee angle measurement, with IMUs placed on the thigh and calf, to manipulate a serious game resembling Flappy Bird. This system can be adapted to the upper limb.

Building upon their previous work presented at the prior congress, Franzo et al. [122] compared a Microsoft Kinect device and Arduino board setup with accelerometer/gyroscope sensors to a reproduction of the same exergame in a mixed reality environment using the HoloLensTM 2. The exergame involved pointing and reaching exercises to enhance upper-limb control during daily activities. According to the authors, the Kinect-based prototype reliably tracks subjects’ movements and kinematic quantities, offering a larger work area compared to the HoloLens. However, the Kinect has lower acquisition frequency and accuracy. The HoloLens 2, with a more restricted work area, allows for more realistic movement training.

The implementation of wristbands as controls for serious games in VR has been investigated to enhance upper-limb function in children with brain injuries [123,124]. Similarly, Jurioli et al. [125] adopted a comparable approach by combining VR with IMU-based wearables as gamepads composed of two IMUs to determine arm orientation (see Table 5).

v. Post-Operative Rehabilitation:

Given the wide applicability of IMUs, IMU utility in post-operative scenarios was also investigated. This is evidenced by the pilot study of Muhlestein et al. [126], demonstrating the capability of activity trackers, such as accelerometers, to measure natural arm movements in children undergoing nerve reconstruction for neonatal brachial plexus palsy (NBPP) over extended follow-up periods. Differently, Zucchi et al. [127] studied wrist ROM recovery post-surgical treatment for distal radius fractures, employing an IMU, and compared outcomes between Kirschner wire fixation (KWF) and volar plate fixation (VPF) with screws. Volar locking plate fixation was found to be comparable to percutaneous fixation for distal radius fracture treatment. In another instance, Yanquez et al. [128] explored the association between vascular surgery results and an upper-extremity function method, utilizing IMU-based wearables on the bicep and forearm.

Also in a post-operative experiment, IMUs have proven effective in objectively monitoring limb recovery following breast and axillary surgery, a task previously reliant on subjective questionnaires, thereby enhancing comparability [129]. Also in the post-operative breast cancer treatment, Vets et al. [130] developed a study integrating IMU-based accessories, cameras, and ML to investigate upper-limb function with post-operative mobility impairment. Two wristbands and video footage served as ground truth data, with a comparative analysis conducted between a machine learning model and a count threshold method (see Table 6).

#### 4.2.2. EMG

Regarding EMG sensors, Feldner et al. [131] conducted a qualitative study involving stakeholders to assess its utilization in neurorehabilitation practice. Healthcare professionals perceive the use of this technology as beneficial for motivation and acquiring objective data. However, the authors emphasized the importance of accessibility and adaptability through intuitive, comfortable, and cost-effective implementations. Many of these characteristics align with the development of most wearable sensors, as evidenced throughout this review.

To jointly reconstruct the entire muscular-kinematic state of the upper limb, Bonifati et al. [132], based on a theoretical solution proposed earlier, developed an undersensorized system using two IMUs and eight surface EMGs electrodes for the same purpose. Through this solution, they were able to jointly reconstruct all 17 degrees of freedom (five joints, twelve muscles) of the upper-limb musculoskeletal state. It is important to emphasize that although this study combines both IMUs with EMGs sensors, it was classified on this category due to the particular interest of the work in EMGs.

Specifically, regarding elbow function, Rahman et al. [133], utilizing EMG signals from the biceps brachii muscle using a three-channel wearable sensor, post-processed by machine learning methods called k-nearest neighbors (k-NN), determined the angle of the elbow (at 0°, 30°, 60°, 90°, and 120°). In a similar application, Mendez et al. [134] conducted a comparative study and numerous methods to calculate finger angle through EMG sensory data. In their study, the authors compared two DL with a standard state-of-the-art decoding technique, determining that both DL conditions (RAW and FFT) perform better.

Regarding the complementarity of EMG with ML methods, Salinas et al. [135] conducted a study comparing several ML algorithms on the classification of 26 ADLs using EMG recordings from the forearm.

In the context of upper-limb rehabilitation to address common secondary injuries and training fatigue, Zhao et al. [136] introduced a wearable device that integrates the combination of EMG and ECG sensors. Additionally, they presented a software platform aiding data analysis, enabling real-time monitoring of hand activities and individuals’ physiological states during training, with the potential for healthcare applications. EMG units were also utilized in distinguishing healthy patients from those with motor issues, specifically patients with elbow trauma [137].

To predict movement intentions for triggering prostheses in amputees, a linear discriminant analysis (LDA) has proven efficient for processing EMG signals. However, in the case of post-stroke patients, there are synergies consisting of involuntary flexion, which can influence the use of LDA, which may be integrated with automated physiotherapeutic methods using exosuits and/or exoskeletons. Considering this doubt, Kopk et al. [138] evaluated whether it is possible to use LDA to make this prediction through myoelectric signals and found promising results. In line with the work of Kopk et al. [138], Merlo et al. [139] developed an algorithm to detect involuntary muscle activity through EMG data. Still, from the perspective of an EMG signal analysis and associated noise, Teh and Hargrove [140] developed an EMG signal filter for upper-limb-prosthesis activation, which is also applicable to, for example, exoskeleton activation. This consisted of developing a supervised denoising variational autoencoder that learns representations of wrist and hand movements that are continuous. The authors showed that this latent space can be used to build noise-resistant classifiers that are significantly more accurate than current state-of-the-art classifiers.

Differently, targeting hand tremor symptoms, Baraka et al. [141] introduced a novel measurement set comprising combined IMU and surface EMG sensors aimed at gathering clinical information from patients diagnosed with PD. The measurements indicate that EMG sensors on the forearm outperform those on the bicep in classifying movement abnormalities. Researchers constructed six machine learning classifiers for the automatic classification of Parkinson’s tremor, including decision tree (DT), linear LDA, KNN, support vector machine (SVM), boosted tree, and bagged tree classifier, which would turn out to be the method with the best results.

**Figure 10:**
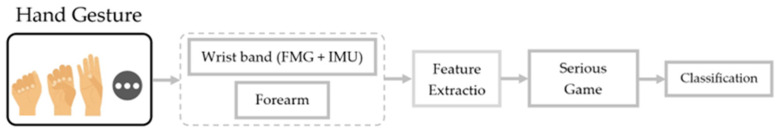
Wearable multimodal rehabilitation utilizing serious games involves extracting kinematic data. Relevant features are identified and input into classification algorithms to predict movements that serve as inputs for the game.

Another work in the field of sensory fusion emerges from Song et al. [142], who developed a movement classification system using a bracelet with an IMU and an FMG and a forearm band with an EMG. This system integrated a serious game in which each detected/classified movement corresponded to an action in the game (see Figure 10).

As identified throughout our review, EMG signals are widely associated with hand pattern identification. Zhou et al. [143] associated this aspect with the development of wearable hand robots and acknowledges that EMG-based pattern recognition performance remains unsatisfactory. Thus, the authors proposed decision fusion methods that combine EMG features and kinematic features (Leap Motion) for hand pattern recognition.

The practice of sensory acquisition through EMGs is commonly associated with wet (gelled) electrodes. However, Abass et al. [144] took a different approach. They developed a dry sensor through 3D printing and validated it for gesture recognition.

The continuous dissemination of this type of body-worn sensors brings with it the challenge of processing substantial amounts of data. With this problem in mind, within the scope of EMG sensors, Kanoga et al. [145] proposed a subject-to-subject transfer framework that uses information available from other people (source) based on ML techniques (see Table 7).

#### 4.2.3. Additional Sensors

Beginning with the out-of-the-box technologies, the starting point of this analysis is the work of Lee et al. [146], who developed a study about a wearable based on an RFID system to monitor hand usage in individuals with upper-limb paresis. The research explores an innovative approach that utilizes RFID technologies to quantify the amount of hand use. The system consists of a wrist-worn RFID reader and a small passive tag placed on an artificial fingernail. Furthermore, a machine learning-based data analysis pipeline is introduced, which processes the backscattered RF signal to estimate the amount of hand use. Inspired by the same concept, Bharadwaj R. and Koul S. [147] characterized the ultra-wideband channel signal (4–8 GHz) through a wearable antenna placed on people’s wrists while they performed a series of exercises. The purpose of this work was to characterize the signal under different conditions; however, the results suggest that it could also be a valid solution like that of Lee et al. [146] presented earlier.

Differently, Yamamoto et al. [148] used a ring-shaped wearable device to measure upper-limb and finger usage simultaneously. The ring-shaped wearable device can measure hand movements and estimate the flexion angle of each finger through light-emitting diode/phototransistor and an IMU.

In the work of Cisnal et al. [149], a device for biocooperative control in neuromotor rehabilitation was developed and was composed of the already explored IMU and EMG sensors as well as GSR, ECG, and SKT sensors. The authors developed such a device with a focus on being highly versatile and low-cost. The system was tested in two scenarios: first, in an upper-limb-rehabilitation virtual reality-based exergame where hand and arm movements are recognized using EMG and IMU data, respectively; second, in the adaptive assistive control of a wrist rehabilitation robot, adjusting assistance levels based on the patient’s physiological state and motor performance using GSR, ECG, and SKT data.

Focusing on the physiological response of patients to a physiotherapeutic task, Badesa et al. [150] developed a wearable of the accessory typology to evaluate the physiological response of patients while establishing a human–machine interface during treatments. The sensory units were EEG and EoG, and the physiological data were HRV and SCL. EEG control is associated with a higher level of stress (associated with a decrease in HRV) and mental workload (associated with a higher level of SCL) when compared to EoG control.

Through the implementation of resistive strain sensors, Ogata et al. [151] developed a wearable sensor that can acquire detailed motion information of patients for remote rehabilitation applications. To this end, 12 strain sensors were attached around the shoulder girdle, and it was possible to obtain detailed motion information of the upper limbs in combination with the IMU.

Other types of deformation sensors found throughout the literature were optical waveguides [152]. These units were evaluated with a carbon fiber layer to constrain the stretching of the optical waveguide, consequently pressing deformation partly and allowing for bending deformation. From the test results, it was concluded that the sensitivity of the proposed sensor to bending is three times higher than that of the typical optical waveguide sensor. This was evaluated in a scenario where it was used as a band on the forearm.

Another less common sensory unit we encountered during the review was barometric pressure sensing. In [153], a wristband composed of a matrix of 10 sensors of this nature was proposed. The purpose of this would be gesture recognition and finger angle estimation with the assistance of ML methods. Another uncommon sensor approach that emerges as an alternative to EMGs for gesture recognition are A-mode ultrasound (US) sensors. Yang et al. [154] conducted a comparative study for these two mentioned sensory units, concluding that A-mode US outperforms EMGs in gesture recognition accuracy, robustness, and discrete force estimation accuracy. In opposition, the authors stated that EMGs is superior to US in continuous force estimation accuracy and ease of use in force estimation.

FMG sensors, this type of sensory unit has been used to capture muscular activity, which serves purposes in human–machine interface applications as well as movement monitoring. In an effort to evaluate this sensory unit, Xiao and Menon [155] identified the minimum sampling frequency needed for recording upper-limb FMG signals without sacrificing signal integrity.

In the field of recognizing human intention for robotic applications, Mariani et al. [156] developed a pressure sensor composed of two piezoresistive units for this purpose. Additionally, Stefanou et al. [157] presented an alternative to EMGs. Their solution consisted of a tactile arm brace (TAB) composed of 8 force-sensitive resistive (FSR) sensors. Using TAB data, machine learning algorithms achieved a classification accuracy of 99%, comparable to a similar commercial intent recognition system based on surface electromyography (EMGs) detection. In contrast to these two aforementioned works, Krausz et al. [158] took the opposite direction by complementing EMGs with gaze tracking glasses. Their proposal involved training two Support Vector Regressors (SVRs) using EMG sensory data to predict hand position, and a Kalman filter-based approach was used to fuse these estimates with a prediction based on the relationship between gaze shifts and arm motion. Fusing gaze and EMG produced higher accuracy position estimates than using EMG alone.

Another common solution described in the literature relies on optical tracking sensors. One of the identified applications, in the field of motor rehabilitation, was their use in synergy with VR from a serious gaming perspective [159]. On the other hand, in the work of Wang et al. [160], this technology was used to predict three-dimensional movements of multiple joints in the human upper limb, focusing on post-stroke rehabilitation. It served as a sensory unit to feed a Deep Neural Network (DNN) based on a simplified kinematic model.

As an alternative to conventional EMG for measuring muscle activity, Meagher et al. [161] conducted a comparative study between electromyography and mechanomyography. The study found that MMG sensory units, which record the mechanical activity of muscles by detecting surface oscillations, provide reliable signals regarding the timing of muscle activity onset, comparable to the reliability of EMG signals.

Lastly, there are several commercially available solutions at affordable costs that have a direct impact on people’s lives. Krisshman V. and Rewale H. [162] extrapolated the basic benefits of a Xiaomi MI fitness band to real and serious preventive health applications. For that, the authors investigated the potential of PPG and IMU units. These authors concluded that shoulder pain significantly increases the energy expenditure among manual wheelchair users and hence should be addressed before wheelchair use for the prevention of injuries (see Table 8).

#### 4.2.4. Actuators

This section is focused on acting elements, such as vibrators and myoelectric stimulation devices. Concerning actuators through vibration, these serve two main purposes in motor rehabilitation: as stimulation or reminders. Regarding reminder application, Signal et al. [165] suggested the use of wrist-worn devices to promote increased usage of the affected arm in post-stroke patients. Additionally, it was utilized to measure the association of upper-limb movement with the occurrence of haptic nudge reminders to move the affected UL in 20 people undergoing inpatient rehabilitation. The results showed an increase equivalent to a 32% rise in the average movement of the affected arm. Further works in this “Remind-to-Move” perspective, such as that of Wei et al. [166] and Mayrhuber et al. [167], followed the same principle of utilizing wearables with vibration stimulation to remind subjects to move the affected arm.

**Figure 11:**
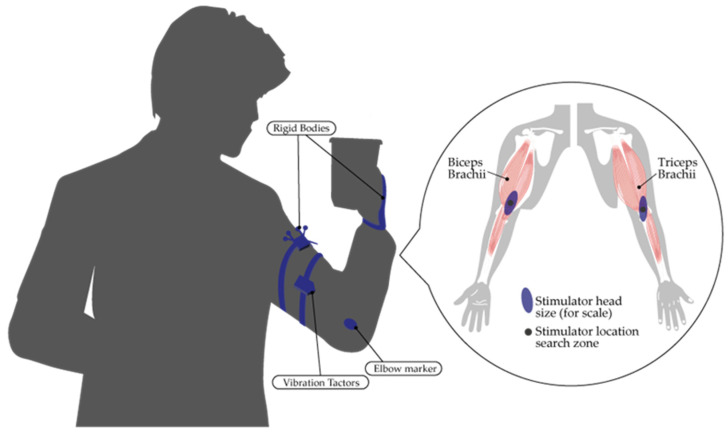
The stimulation system proposed in the work by Ferrari et al [169].

On the other hand, Wang et al. [168], leveraging the benefits of vibration, recognized its potential to enhance and synchronize muscle recruitment while fostering muscle strength and endurance. Consequently, the authors presented a wearable system that delivers vibration-based muscle activation for upper-limb function rehabilitation, extending the reach of rehabilitation efforts beyond clinical settings. Similarly, in [169], it was demonstrated that illusion-inducing vibrations can be used as augmented proprioceptive sensory feedback to improve the motor performance of stroke patients (see Figure 11). Inspired by the previous works, Pennington et al. [170] assessed the potential of vibration technologies on reach and grasp activities, concluding that improvements were more pronounced for grasping than for reaching. Furthermore, in the field of vibration stimulation, this functionality was also verified for haptic feedback purposes. In [171], an active wearable via vibration was introduced to provide feedback on upper-limb extremity velocity. Additionally, Mortaza et al. [172] studied muscle-tendon vibration over the wrist flexors and extensors, finding that the vibration group had faster reactions and superior performance in the transfer condition.

Regarding electrical muscle stimulation, Choudhury et al. [173] conducted a study in this area, complementing it with auditory clicks. They involved 95 post-stroke patients randomized into three groups, utilizing a wearable device delivering stimulation for at least 4 h daily over 4 weeks. Significant improvements in upper-limb function were observed in the paired stimulation group, particularly in grasp function, suggesting meaningful gains in hand function for stroke survivors. Differently, Ward et al. [174] studied the effect of electrical stimulation on the performance of standardized manual gestures. This technology of muscle stimulation was further integrated into a solution constantly in symbiosis with EMG sensory units. Crepaldi et al. [175] developed a device that integrates these mentioned technologies, managing the stimulation level based on muscle activity detected via EMG. In a similar concept, Yong and Sai [176] developed a myoelectric stimulation system driven by sensory data from EMG but involving a human-to-human interface (HHI) between patient and therapist. The uniqueness lies in the dynamic stimulation resulting from the therapist’s feedback, as the therapist had EMG sensors attached to measure the intensity of their movements to vary the stimulation in the patient accordingly. Similarly, Kim et al. [177] combined an IMU sensor unit in a wristband to analyze upper-limb tremors with an electrode to modulate/attenuate tremors through peripheral-nerve electrical stimulation. This system showed a significant reduction in both tremor frequency and power (Table 9).

## 5. Discussion and Future Perspectives

**Figure 12:**
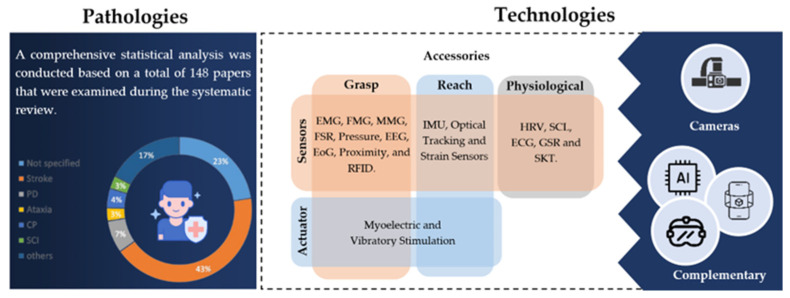
Contributions from the conducted review, including the characterization of the pathologies explored in the studies as well as the main technologies addressed.

The introduction of wearables into people’s daily lives has significantly increased in recent years. If initially they were primarily used for tracking physical activity parameters, mainly for recreational purposes, with their evolution, the applicability of these devices has expanded to various uses. With their impacts becoming more significant and increasingly relevant to continuous health promotion, they have started gaining traction among healthcare professionals. In the field of motor rehabilitation, for example, in the past, the process of motor recovery was limited exclusively to the clinical environment. Nowadays, it is increasingly possible to extend the treatment and monitoring of patients’ motor abilities to the home setting, both mechanically and physiologically. Based on these two pillars, mechanical or physiological responses, our discussion is structured around the technologies that integrate wearable systems and their applications to assess the impacts they have on patients’ lives (see Figure 12).

Technologically, in terms of monitoring, the systems identified in the review can integrate various sensory units and/or actuator essential for the recovery and monitoring of upper-limb motor functions. In terms of motor response, it can be categorized as the capacity to reach and grasp, where IMU and EMG sensors play a dominant role, according to our review. Although they were the most prominent sensory units, other solutions can be found in the literature. For assessment reach capacity, deformation sensors (capacitive, resistive, and optical waveguide types) were often associated with joint reconstruction performance. In this area of strain sensors, there is a recognized need to address their susceptibility to interference from changes in temperature and humidity, particularly when attached directly to the body. Although these aspects were not the primary focus of this study due to the specific requirements defined in the methods section, it is important to highlight that strain sensors have been the subject of recent investigations aimed at overcoming these challenges. Various studies have explored approaches to enhance sensor stability under variable environmental conditions [180,181]. These contributions highlight the importance of conducting a literature review in this area to deepen understanding and drive advancements in the field. On the other hand, for evaluation of grasp capacity, alternatives included MMGs, FMG, FSR, A-mode US, or even optical waveguides. Regarding physiological response, an analysis can be conducted based on patients’ reactions to specific treatments and physiotherapy exercises by measuring parameters such as skin temperature and conductivity, EEG, among others [150]. Additionally, from the perspective of promoting motor rehabilitation, actuators play a crucial role in localized muscle or nervous stimulation. They can serve as reminders for performing physical activities or even assist in muscle activation and the reduction of tremors through localized vibratory and micro-electrical stimuli. In some cases, in the literature, active elements are combined with sensory units that serve as stimulus managers depending on the level of muscle activation recorded.

All these described technologies have been integrated into wearable systems designed for various purposes, applications, and impacts on patients’ lives. These devices have evolved to such an extent that they can now compare to some gold-standard tools and have opened new possibilities for continuous health promotion that were previously unattainable. As identified by some studies, motor skills acquired in clinical physiotherapy do not always translate into practical results in patients’ daily lives. Thus, these systems play a crucial role in remote patient monitoring aids in decision-making for patient treatment. These devices have evolved to such an extent that they can now compare to some gold-standard tools and have opened new possibilities for continuous health promotion that were previously unattainable. This concept also led to the introduction of egocentric cameras that significantly impact the monitoring of patients with upper-limb disabilities, whether due to a pathology or post-operative conditions, by observing how they interact with everyday objects. Beyond recovery monitoring, these systems are also essential for tracking motor degradation due to pathological reasons or increased frailty with aging.

Throughout our review, numerous wearable systems were introduced, encompassing a wide range of technologies, from passive components (sensors) to active ones (actuators). Given their critical importance and impact on the reliability of the wearable systems they are integrated into, it would be highly beneficial for future studies to focus more extensively on the characteristics of these components. This would enable readers to perform comparative analyses of similar wearable systems in the literature. However, as these components (sensors and actuators) are integrated into more complex wearable systems designed for specific diagnostic purposes, effective comparisons and quality validation would necessitate the standardization of gold-standard tools as validation metrics for these systems. As observed in Section 4.2.1. (ii) (IMUs, Comparison with Other Methods), only a limited number of studies have implemented this practice, highlighting a gap that should be addressed in future research.

Wearable systems are often combined with AI processing methods and complementary technologies. AI methods play a critical role in the post-processing and analysis of sensory data, aiding healthcare professionals in decision-making through their ability to identify patterns that classify patients’ health states or predict their progression. Complementing wearable systems with technologies such as VR, MR, and AR, which intersect with gaming concepts where wearables serve as game controllers, enhance patient adherence to more immersive and tailored treatment forms.

While the concept of wearable systems has already advanced motor recovery, there is still a long way to go. Most of the research on this topic is heavily focused on monitoring and decision-making rather than being a full-fledged therapeutic tool that can be used at home to promote continuous treatment. Moreover, there is a lack of contextualization regarding daily activities within monitoring and physiotherapy procedures, which is also reflected in the commercial offerings available. As observed throughout our review, the primary commercially available accessories are predominantly smartwatches and smart bands, likely due to their user-friendly nature and relatively simple design, which helps keep costs manageable. While these devices effectively monitor reach capacity, they fall short in capturing grasp capability. Given these considerations, a promising approach could be to incorporate sensors into everyday objects and utensils. This approach would enable the monitoring of patients’ interactions with common items in their daily routines without disrupting their usual activities.

Additionally, it is important to highlight that throughout this review, data privacy considerations are rarely emphasized in the majority of studies despite this being a critical factor when discussing wearables that continuously collect patient health data. Given that these devices are frequently viewed as tools for remote medical monitoring, they are required to provide connectivity, which raises heightened concerns regarding data privacy and potential vulnerabilities that could compromise data confidentiality. This issue should be a key focus for future developments in the field.

## 6. Conclusions

This paper reviewed wearable systems in the context of the continuous health of the upper limb specifically in motor rehabilitation, monitoring, and prediction. Numerous studies in this same domain were identified despite presenting highly diverse technological principles. Through a more detailed analysis of accessory-type devices, this study identified numerous pathologies for which proposals could be made, focusing particularly on the context and, more importantly, the sensory and actuation units they integrate. Thus, it is possible to provide readers with a holistic perspective on the evolution of this field and, essentially, to create connections between wearable systems with different purposes and the ability to adapt identified technologies to different realities. For instance, we observe inertial units being used to assess the quality of reach and grasp for post-stroke patients or to evaluate the amplitude and frequency of upper-limb vibration in Parkinson’s patients. One wonders whether some of the systems integrating IMUs and only proposing to address one of these pathologies could not be adapted for the other.

Throughout this work, it is evident that the focus was placed on what we consider fundamental technologies, primarily directed towards hardware development. However, it is important to highlight the significance of complementary technologies. We associate the driver with complementary technologies, as they play a fundamental role in wearable adoption through a more immersive application with VR, MR, and AR or even in the post-processing of sensor data through the implementation of ML and DL methods.

This study clearly demonstrates the potential of wearable technologies as potential medical tools. In a time when there is much talk of decentralization, Web3, among others, it is evident that telerehabilitation applied to motor rehabilitation, monitoring, and/or prediction thereof can also move towards the decentralization of medical units. However, it is important that many of these works be translated into marketable devices to avoid the disproportionality that exists in Europe between the number of published scientific articles and the number of commercialized devices [26].

## Figures and Tables

**Table 1:** List of acronyms mentioned throughout the paper.

Acronyms	Meaning	IMU	Inertial Measurement Unit
ADLs	Activities of Daily Living	KWF	Kirschner wire fixation
AOT	Action Observation Training	LDA	linear discriminant analysis
ARAT	Action Research Arm Test	ML	Machine Learning
AI	Artificial Intelligence	MMG	Mechanomyography
AR	Augmented Reality	MR	Mixed Reality
CP	Cerebral Palsy	NBPP	Neonatal Brachial Plexus Palsy
CNN	Convolutional Neural Network	KNN	K-Nearest Neighbours
DL	Deep Learning	OT	Occupational Therapists
DNN	Deep Neural Network	PD	Parkinson’s Disease
ECG	Electrocardiography	RFID	Radio Frequency Identification
EEG	Electroencephalographic	ROM	Range of Motion
EMG	Electromyography	SCI	Spinal Cord Injury
EoG	Electrooculographic	SKT	Skin Temperature
FMG	Force myography	SVR	Support Vector Regressors
FSR	Force Sensitive Resistive	SVM	Support Vector Machine
FMA	Fugl-Meyer Assessment	TAB	Tactile Arm Brace
FAS	Functional Ability Scale	US	Ultrasound
GSR	Galvanic Skin Response	VPF	Volar Plate Fixation
GM	Gross Movement	VR	Virtual Reality
HRV	Heart Rate Variability	YOLO	You Only Look Once

**Table 2:** Study characteristics for studies for monitoring, diagnosis, and motor dysfunction assessment.

Ref.	Year	Pathology	Technology	Study Goals
[56]	2020	Stroke (Hemiparesis)	Mi Band 3 (Xiaomi Inc., Beijing, China)	Assess asymmetry between affected and unaffected limb
[57]	2019	Stroke (Hemiparesis)	ActiGraphs GT3X+ (ActiGraph, Pensacola, FL, USA)	Assess asymmetry between affected and unaffected limb
[58]	2019	Stroke	outREACH (Custom made wrist-worn)	Impairment determination of the upper limb based on asymmetry
[59]	2020	Stroke (Hemiparesis)	Wrist-worn	Identification and quantification of the level of hemiparesis
[60]	2020			
[61]	2020		Not specified	
[62]	2021			
[63]	2023	CP	ActiGraphs GT3X+ (ActiGraph, Pensacola, FL, USA)	Track and measure gross upper-limb movement
[64]	2021	Stroke (Hemiparesis)	Custom wrist-worn composed by SEN-14001 board (Spark Fun Inc., Niwot, CO, USA) and IMU MPU9250, (InvenSense-TDK Co., San Jose, CA, USA)	Quantify arm use
[65]	2021	Stroke	TENZR^TM^ Neuro Tracker V4 X. (BioInteractive Technologies Inc., Vancouver, BC, Canada)	Assessment of reach and grasp activity
[66]	2019			
[67]	2020	Stroke	IMU network (ST LSM330DLC manufactured by STMicroelectronics, Geneva, Switzerland)	Assessment of reach and grasp activity
[68]	2022	Not specified	Bicep and forearm band (model no specified) and Logitech C270 (Logitech International S.A, Riviera, Switzerland)	Joint angles estimation in real-time during rehabilitation
[69]	2021	Musculoskeletal disorders	17 IMUs MTws (Xsens Technologies, Enschede, The Netherlands)	Joint angles estimation for ergonomic risk assessment
[70]	2023	Not specified	WT901BLECL5.0 (Wit-MotionCompany, ShenZhen, China)	Madgwick filter-based Joint angles
[71]	2020	Not specified	IMU network of BNO055 sensor (Bosch Sensortec GmbH, Stuttgart, Germany)	Joint angles of the shoulder and elbow
[72]	2023	Not specified	Shimmer3 (Shimmer, Dublin, Ireland)	ROM assessment enabling the simultaneous estimation of joint angles and positions
[73]	2023	Stroke	Wristwatch SCW-V2 (The Hong Kong Polytechnic University, Hung Hom, Hong Kong)	Arm ROM measurement
[74]	2021	Hand surgery, stroke, rheumatoid and arthritis	InSense (Arsalis, Louvain-la-Neuve, Belgium)	Upper-limb assessment during Manual Activities of Daily Living
[75]	2023	Stroke	MYO armbands (Thalmic labs, Kitchener, ON, Canada), IMU network and EMG bracelets (models not specified)	Upper-limb assessment during Manual Activities of Daily Living
[76]	2023	Perinatal brain injury (Not specified)	Axivity AX3 (Axivity Ltd., Newcastle, UK)	Assessment scores of infants’ motor activity
[77]	2023	Neonatal brachial plexus palsy	Actigraph GT9X Link (ActiGraph, Pensacola, FL, USA)	Quantification of the duration and magnitude of patient-initiated arm movements
[78]	2019	SCI	ReSense [88]	Assess upper-limb compensation
[79]	2020	Not specified	Custom armband composed by MPU6050 (TDK InvenSense, Tokyo, Japan) and nRF24L01 (Nordic Semiconductor, Trondheim, Norway)	Armband geared towards data transmissibility in the context of mobile medicine
[80]	2022	Stroke	Finger-worn not specified	Reliability of inertial finger registration for treatment customization
[81]	2021	Stroke	Custom upper-limb band (IMU sensor from STMicroelectronics, Geneva, Switzerland))	Quantify hand and wrist motor function
[82]	2020	Ataxia	Wrist-worn composed by MPU9250 (TDK InvenSense, Tokyo, Japan), LPC1768 Microcontroller (NXP Semiconductors, Eindhoven, The Netherlands) and GS2011MIZ (GSI Technology, Sunnyvale, CA, USA); and a Kinect v2 (Microsoft, Seattle, WA, USA)	Diagnosis and monitoring of ataxia progression
[83]	2022	PD	Custom wrist-worn composed by Arduino (Arduino Srl, Turin, Italy) and MPU6050 (TDK InvenSense, Tokyo, Japan)	Monitoring upper-limb tremor detection
[84]	2021	PD	Kinesia One (Great Lakes Neurotechnologies Inc, Independence, OH, USA)	Monitor progression and define the onset of manifestation
[85]	2021	Stroke	Actigraph GT9X (ActiGraph, Pensacola, FL, USA)	Monitoring, and predicting the motor evolution of patients after their first stroke
[86]	2023	Unilateral Spastic CP	Axivity AX3 (Axivity Ltd., Newcastle, UK)	Monitor upper-limb function during AOT
[87]	2023	Dyskinetic CP	XSens MTw Awinda (XSens Technologies, Enschede, The Netherlands)	Detect pathological movements in individuals with Dyskinetic CP.

**Table 3:** Study characteristics for studies that compare other methods.

Ref.	Year	Pathology	Technology	Study Goals
[89]	2022	Not specified	Wave Track inertial system (Cometa Systems, Milan, Italy) and Vicon MX T10S (Vicon Motion Systems, Oxford, UK)	Comparison of a portable inertial sensor system with optical motion analysis to measure shoulder kinematic
[90]	2022	Not specified	XCLR8 IMU (XCLR8 Technologies Private Limited, Singapore) and Eagle digital cameras (Motion Analysis Corp., Rohnert Park, CA, USA)	Alternative to conventional ROM measurement methods
[91]	2022	Stroke	Modified version of the original T’ena sensor [58] and VICON Bonita 10 (Vicon Motion Systems, Oxford, UK)	Accurately measuring movement kinematics during post-stroke motor tasks compared to a gold-standard motion capture system.
[92]	2022			
[93]	2020	Not specified	EXLs3 (Exel srl, Bologna, Italy) and Kinect v2 (Microsoft, Seattle, WA, USA)	Human motion tracking.
[94]	2021	Not specified	IMU network and Kinect v2 (Microsoft, Seattle, WA, USA)	Human motion tracking.

**Table 4:** Study characteristics for studies that apply ML techniques.

Ref.	Year	Pathology	Technology	Study Goals
[95]	2021	Ataxia	Opal (APDM Wearable Technologies, Portland, OR, USA)	ataxia severity assessment
[96]	2020	Stroke	XSens MTw Awinda (XSens Technologies, Enschede, The Netherlands)	upper-limb impairment estimation
[97]	2022	Stroke (hemiparesis)	XSens MTw Awinda (XSens Technologies, Enschede, The Netherlands)	impairment severity estimation
[98]	2021	PD	Opal (APDM Wearable Technologies, Portland, OR, USA)	comparison between upper- and lower-limb inertial data to diagnose PD and determine the stage of the disease
[99]	2019	PD	Custom fingers/wrist-worn composed by Cortex-M3 CPU (STMicroelectronics, Geneva, Switzerland) and LSM9DS (STMicroelectronics, Geneva, Switzerland)	PD stage quantification
[100]	2022	PD	BWT901CL (Wit-MotionCompany (Wit-MotionCompany, ShenZhen, China)	PD diagnosis
[101]	2023	PD	Custom fingers/wrist-worn composed by MPU9250 (TDK InvenSense, Tokyo, Japan) and nRF52832 (Nordic Semiconductor, Trondheim, Norway)	Distinguish Parkinson’s patients
[102]	2022	Not specified	Shimmer3 (Shimmer, Dublin, Ireland)	Classification of the load exerted on the shoulders
[103]	2023	Not specified	APDM Wearable model not specified (APDM Inc., Portland, OR, USA)	Predict pain
[104]	2023	Not specified	STEVAL-STLKT01V1 (ST Microelectronics, Geneva, Switzerland)	Joint prediction
[105]	2022			
[106]	2021	Not specified	STEVAL-STLKT01V1 (ST Microelectronics, Geneva, Switzerland)	Detect elbow movement intention
[107]	2023	Stroke	STEVAL-STLKT01V1 (ST Microelectronics, Geneva, Switzerland)	Motion intention detection
[108]	2019	Stroke	Shimmer model not specified (Shimmer, Dublin, Ireland)	Monitor physiotherapeutic evolution
[109]	2020	Stroke	Shimmer2 (Shimmer Sensing, Dublin, Ireland)	Clinical scores estimation
[110]	2020	Stroke	Smartwatch W270 (LG, Seoul, South Korea)	Identify and log the variety and frequency of rehabilitation exercises performed by the individual
[111]	2021	Stroke	Actigraph GT3X-BT or GT9X-Link (ActiGraph, Pensacola, FL, USA)	Categorization of upper-limb performance
[112]	2019	Stroke	Finger and wrist-worn (Arcus, ArcSecond Inc., San Diego, CA USA)	Hand use during ADLs estimation
[113]	2022	Not specified	Wrist-worn [64]	Comparison of different measures for assessing upper limb
[114]	2022	Stroke (hemiparesis) and Traumatic brain injury	Shimmer2r (Shimmer Sensing, Dublin, Ireland)	Estimation of clinical scores reflecting upper-limb movement quality
[115]	2022	Stroke	ZurichMOVE (ZurichMOVE, Zurich, Switzerland)	Estimation task scores
[116]	2021	Stroke (hemiparesis) and traumatic brain injury	Shimmer2 (Shimmer Sensing, Dublin, Ireland)	Predict and monitor the recovery process and assess the responsiveness to treatment on an individual basis
[117]	2019	Stroke (hemiplegia)	wrist-worn (not specified)	Motion recognition
[118]	2021	Stroke	Custom Arm band composed by MPU9250 (TDK InvenSense, Tokyo, Japan), Arduino Nano V3 (Arduino Srl, Turin, Italy) and Wireless Transmitter HC-06	Motion recognition of upper-limb exercises and improvement of recognition performance
[119]	2019	Not specified	Smartwatch W270 (LG, Seoul, South Korea)	Optimal size estimation of the time window for classifying real-time motions

**Table 5:** Study characteristics for gamified rehabilitation studies.

Ref.	Year	Pathology	Technology	Study Goals
[120]	2020	Not specified	GC-Rebot (custom hand band composed by IMU, radio transceiver, and MCU) and XSens MTw Awinda (XSens Technologies, Enschede, The Netherlands)	Physiotherapeutic gamepad to control via gestures a car within a maze
[121]	2020	Neuromuscular and musculoskeletal diseases	XSens MTw Awinda (XSens Technologies, Enschede, The Netherlands)	Physiotherapeutic gamepad to manipulate a serious game
[122]	2022	Ataxia	Microsoft Kinect One (Microsoft, Seattle, WA, USA), Microsoft HoloLens 2 (Microsoft, Seattle, WA, USA), and a custom hand sensor	Pointing and reaching serious game
[123]	2021	CP	RAPAEL Smart Kids (Neofect Co., Ltd., Gyeonggi-do, Republic of Korea)	Physiotherapeutic gamepad for serious games in Virtual Reality
[124]	2023			
[125]	2020	Stroke, Cognitive deficit and musculoskeletal diseases	Custom arm band composed by Arduino Nano (Arduino Srl, Turin, Italy), MPU6050 (TDK InvenSense, Tokyo, Japan) and HC-05 Bluetooth Module	Physiotherapeutic gamepad for serious games in Virtual Reality

**Table 6:** Study characteristics for post-operative rehabilitation studies.

Ref.	Year	Pathology	Technology	Study Goals
[126]	2022	Neonatal Brachial Plexus Palsy	GT9X-Link (ActiGraph, Pensacola, FL, USA)	Activity trackers to measure natural arm movements
[127]	2020	Distal radius fractures	hand band model not specified (Fisiocomputer, Rome, Italy) and BTS Freeemg 300 (BTS Bioengineering, Garbagnate Milanese, Italy)	comparison of results between KWF and VPF with screws
[128]	2020	Not specified	Arm band model not specified (BioSensics LLC, Newton, MA, USA)	Study of the association between vascular surgery results and an upper-extremity function method
[129]	2021	Breast cancer	Axivity AX3 (Axivity Ltd., Newcastle, UK)	Monitor limb recovery following breast and axillary surgery
[130]	2023	Breast cancer	Actigraph GT3X-BT (ActiGraph, Pensacola, FL, USA) and Sony FDR-AX33 (Sony, Tokyo, Japan)	Study of the upper-limb function in post-operative breast cancer patients

**Table 7:** Study characteristics for EMG studies.

Ref.	Year	Pathology	Technology	Study Goals
[131]	2020	Stroke	MC10 BiostampRC (Lexington, MA, USA), Thalamic Labs My Armband (Kitchener, ON, Canada), Delsys Trign (Natick, MA, USA); and a lab-designed prototype Epidermal Sensor System (Austin, TX, USA, patent pending),	Assess EMG utility in neurorehabilitation practice
[132]	2023	Not specified	Delsys Bagnoli EMG system (Delsys Inc., Natick, MA, USA), the Xsens MTw Awinda (XSens Technologies, Enschede, The Netherlands) and custom IMU based wearable system	Muscular-kinematic reconstruction of the upper limb
[133]	2021	Not specified	Shimmer3 EMG (Shimmer, Dublin, Ireland) and EMG electrodes	Determine the angle of the elbow
[134]	2021	Amputees	EMG Noraxon Delsys (Natick, MA, USA) and LabJack (LabJack Corporation, Lakewood, CO, USA)	Finger angle regression
[135]	2022	Not specified	Biometrics EMG (Biometrics Ltd., Newport, UK)	Comparison of several ML algorithms on the classification of 26 ADLs
[136]	2020	Not specified	Custom ECG device composed by na), read using the STM32L152 chip (STMicroelectronics, Geneva, Switzerland), BMD101 A/D converter (NeuroSky Company, Wuxi, China), electrodes and BLE module. Custom EMG deice composed by STM32L152 chip (STMicroelectronics, Geneva, Switzerland), AD8221 amplifiers (Analog Devices, Wilmington, MA, USA) and BLE module.	Wearable device to obtain accurate signals during robotic glove-assisted training
[137]	2019	elbow trauma	Trigno Wireless system (Delsys Inc., Natick, MA, USA)	Distinction between healthy and elbow trauma patients
[138]	2019	Stroke	Delsys 16 channel Bagnoli (Delsys Inc., Natick, MA, USA) and a load cell 45E15A (JR3 Inc., Woodland, CA, USA)	Movement intention prediction
[139]	2023	Motor Neuron Lesion	Mini Wave Plus, (Cometa Systems, Milan, Italy)	Detect involuntary muscle activity
[140]	2021	Not specified	Arm band with six electrodes (Motion Control Inc., Salt Lake City, UT, USA) and Ag/AgCl electrodes (Bio-Medical Instruments, Shenzhen, China)	EMG signal filter for upper-limb-prosthesis activation
[141]	2019	PD	IMU and EMG Shimmer (Shimmer, Dublin, Ireland)	Clinical information acquisition system for PD patients
[142]	2022	Stroke	Custom 6 EMG (Trigno Wireless EMG System, MAN-012-2-6, Delsys Inc., Natick, MA, USA) forearm band and eight barometric (MPL115A2, Freescale Semiconductor Inc., Austin, TX, USA) plus one IMU Wristband (BNO055, BOSCH Inc., Baden-Württemberg, German)	Movement classification to control a serious game
[143]	2021	Stroke	Trigno wireless EMG system (Delsys Inc., Natick, MA, USA) and infrared motion sensor (Leap Motion Inc., San Francisco, CA, USA)	EMG-based pattern recognition
[144]	2019	Stroke	Shimmer with custom dry 3D printed electrodes (Shimmer, Dublin, Ireland)	Development of a dry sensor through 3D printing
[145]	2021	Not specified	Myo Gesture Control Armband (Thalmic Labs, Kitchener, ON, Canada)	EMG data transfer framework between subjects based on ML techniques

**Table 8:** Study characteristics for additional sensors studies.

Ref.	Year	Pathology	Technology	Study Goals
[146]	2019	Paresis	Custom fingernail (RFID) and custom wrist worn (M6E-M RFID reader kit, ThingMagic, Bedford, MA, USA)	Monitor hand usage
[147]	2019	Not specified	Not specified	Characterization of the ultra-wideband channel signal during exercise performance
[148]	2023	Stroke (hemiplegia)	Custom finger band composed by LED SFH 4550 (Osram Opto Semiconductors, Regensburg, Germany) and Phototransistor SD5410 (Honeywell International Inc., Charlotte, NC, USA) and wristband composed by Adafruit Feather M0 Adalogger (Adafruit Industries, New York, NY, USA)	Hand movement measurement and estimation of finger angle flexion
[149]	2023	Not specified	Custom board composed by IMU ICM-20948 (InvenSense, San Jose, CA, USA), ECG AD8232 (Analog Devices, Wilmington, MA, USA), EMG MCP3912 (Microchip Technology, Chandler, AZ, USA), GSR LM324 (Texas Instruments, Dallas, TX, USA), SKT MLX90614 (Melexis, Tessenderlo, Belgium), MCU TMS320F28069M (Texas Instruments, Dallas, TX, USA), BLE CC2650 (Texas Instruments, Dallas, TX, USA) and MCP73831 (Microchip Technology, Chandler, AZ, USA).	Adaptive assistive control of a wrist rehabilitation robot based on the patient’s physiological state.
[150]	2019	Not specified	Tobii Glasses (Tobbi, Stockholm, Sweden), e Enobio 8 (Neuroelectrics, Barcelona, Spain), BioHarness 3 (Zephyr Technology, Washington, DC, USA) and Shimmer 3 GSR+ (Shimmer, Dublin, Ireland)	Monitor physiological response of patients while establishing a human–machine interface
[151]	2022	Not specified	XSens MTw Awinda (XSens, Enschede, The Netherlands) and a custom system composed by an IMU and a network of strain sensors [163]	Monitoring shoulder motion
[152]	2023	Not specified	Custom Optical waveguides composed by two elastomers, a polyurethane elastomer corewith a higher refractive index (Smooth-on Inc., Macungie, PA, USA), a LED TSHA4400, Vishay Semconductors, Malvern, PA, USA), photodiode SFH229 (arm-OSRAM AG, Premstaetten, Austria)	Carbon fiber layer to constrain the stretching of the optical waveguide
[153]	2019	Not specified	Custom wristband composed by 10 modified barometric pressure sensor units (TakkTile, New York, NY, USA)	Wristband to recognise gestures and to estimate finger angle
[154]	2020	Stroke and Amputee	A-mode US transducers (model not specified) and Biometrics EMG (Biometrics Ltd., Newport, UK)	Comparative study between A-mode US and EMG sensors in gesture recognition
[155]	2019	Not specified	Custom forearm and distal strap composed by 8 FSR402 FMG sensors (Interlink Electronics Inc., Camarillo, CA, USA)	Minimum sampling frequency needed for recording upper-limb FMG signals
[156]	2022	Not specified	Custom arm strap composed by a piezoresistive unit fixed on a 3d printed structure, and National Instrument USB-6003 for data acquisition (National Instrument, Austin, TX, USA)	Recognizing human intention
[157]	2019	Stroke	Tactile arm brace (TAB), composed by 8 FSR sensors (model not specified) and use an Arduino (Arduino Srl, Turin, Italy) to acquire data.	Recognizing human intention
[158]	2020	Stroke and Amputee	Telemyo dts (Noraxon, Scottsdale, AZ, USA) and SMI Eye Tracking Glasses (SensoMotoric Instruments, Teltow, Germany)	Gaze and EMG fusion to predict hand position
[159]	2020	CP and Developmental Dyspraxia	Haptic Device [164], Oculus Rift VK2 (Meta Platforms, Inc., Menlo Park, CA, USA) and OptiTrackTM V120 Trio (NaturalPoint, Inc. DBA OptiTrack, Corvallis, OR, USA)	Physiotherapeutic efficacy of combining VR and wearable haptic devices.
[160]	2021	Stroke	Vicon Motion Capture (Vicon Motion Systems, Ltd., Oxford, England)	3D movement prediction of multiple joints
[161]	2020	Stroke	Biometrics EMG (Biometrics Ltd., Newport, UK) and a custom device composed by Microphone SPU1410 (Knowles, Itasca, IL, USA).	Comparative study between electromyography and mechanomyography for measuring muscle activity
[162]	2020	SCI	Xiaomi MI Band Version 2 (Xiaomi Inc., Beijing, China)	Impact of shoulder pain on energy expenditure in manual wheelchair users.

**Table 9:** Study characteristics for actuator studies.

Ref.	Year	Pathology	Technology	Study Goals
[165]	2020	Stroke	BuzzNudge 310-103 (Precision Microdrives Ltd., London, UK)	Increase the usage of the affected arm in post-stroke patients.
[166]	2019	Stroke	Wristwatch SCW-V2 (The Hong Kong Polytechnic University, Hung Hom, Hong Kong)	Increase the usage of the affected arm in post-stroke patients.
[167]	2023	Stroke	ARYS^TM^ me|tracker and ARYS^TM^ pro|tracker (Tyromotion Gmbh, Graz, Austria)	Increase the usage of the affected arm in post-stroke patients.
[168]	2022	Stroke	Custom vibration device, FoVi, composed by four vibration motor pods	muscle recruitment
[169]	2021	Stroke	Vibrasens VB200, (TechnoConcept, Manosque, France)	muscle recruitment
[170]	2023	Stroke	TheraBracelet [178]	Improvements for reach and grasp capability more pronounced for grasping
[171]	2023	CP	TELOS [179]	feedback on upper-limb extremity velocity
[172]	2023	Stroke	Two-point discrimination 16022 (Lafayette Instrument, Lafayette, IN, USA), Touch-TestVR Sensory Evaluators: SemmesWeinstein Monofilaments (North Coast Medical, Morgan Hill, CA, USA) and Eccentric Rotating Mass motor (model not specified)	enhance reactions and performance
[173]	2020	Stroke	Electrical stimulator with a constant current and 220 V compliance (model not specified), Dynamometers Power and Pinch Grip (Biometrics Ltd., Newport, UK) and Electrogoniometer SG75 (Biometrics Ltd., Newport, UK)	Significant improvements in upper-limb function particularly in grasp function
[174]	2020	Stroke	Custom stimulation system, Microsoft Kinect v2 (Microsoft, Seattle, WA, USA), Raspberry Pi (Raspberry Pi Foundation/Raspberry Pi Trading Ltd., Cambridge, UK),	reliably generate specific target gestures on healthy users
[175]	2021	Stroke (Hemiplegia)	FITFES, custom necklace layout system composed by EMG amplifier AD8326 (Analog Devices, Wilmington, MA, USA), STM32L476 (STMicroelectronics, Geneva, Switzerland), Bluetooth BGM13P (Silicon Labs, Austin, TX, USA), HVPS Flyback regulator (not specified), IMU (not specified), Functional Electrical Stimulation eletrodes (Axelgaard, Fallbrook, CA, USA), EMG electrodes (MedicoTest A/S, Glamsbjerg, Denmark), LED Indicators (not specified).	EMG-controlled electrical stimulation system.
[176]	2022	Stroke and SCI	Custom neuromuscular electrical stimulation composed by EMG, accelerometer, LEDs and A/D converters (models not specified)	Human-to-human management stimulation signals between the patient and therapist.
[177]	2020	Essential tremor	Custom wristband composed by IMU LSM303D (STMicroelectronics, Geneva, Switzerland), microcontroller CC2510 (Texas Instruments, Dallas, TX, USA), Wireless Transceiver (not specified), Voltage-mode Stimulator Circuitry—(custom-built, not specified), battery Charger LTC4054 (Analog Devices, San Jose, CA, USA) Boost Converter LM27313 (Texas Instruments, Dallas, TX, USA), Electronic Switches (not specified), Variable Resistor AD5162 (Analog Devices, Wilmington, MA, USA) Surface Electrodes (Syrtenty, São Paulo, Brazil).	Tremors attenuation
